# Clinical Decision Support for Adult Enteral Nutrition: Literature Overview and Preliminary Hospital‐Based Evaluation

**DOI:** 10.1111/jep.70612

**Published:** 2026-10-12

**Authors:** Panos Papandreou, Alexandra Foscolou, Aristea Gioxari, Paraskevi Detopoulou

**Affiliations:** ^1^ Department of Nutrition IASO Hospital Athens Greece; ^2^ Department of Nutritional Science and Dietetics School of Health Sciences, University of the Peloponnese Antikalamos Kalamata Greece

**Keywords:** adults, clinical decision support system, computer software, enteral nutrition, enteral supplements, nutrition assessment

## Abstract

**Rationale:**

Clinical Decision Support Systems (CDSS) are increasingly used to enhance medical decision‐making, including nutrition support.

**Objective:**

This study aimed to narratively review CDSS tools for enteral nutrition (EN) and to preliminarily evaluate a locally developed internal‐use CDSS designed to calculate patients' nutritional requirements and assist in the selection of EN and oral nutritional supplements (ONS) in adult hospitalized patients.

**Methods:**

Literature search was conducted in PubMed to identify existing CDSS tools used for assisting EN. Additionally, the internal‐use CDSS was pilot‐tested in 27 adult inpatients admitted to a private hospital in Athens, Greece. Differences in the time required to prepare the manually derived EN/ONS plan and the CDSS‐derived plan were recorded. Discrepancies between the two approaches were assessed.

**Results:**

Fourteen studies were identified. Although the results were inconsistent, the application of a CDSS seems to reduce calculation time, increase dietitian referrals, initiate faster enteral feeding, improve nutrient delivery, decrease nutritional risk and was associated with fewer feeding‐related medical errors. In the pilot‐test of the internal‐use CDSS, the time required for nutrition planning decreased from 9.01 ± 0.21 min to 4.01 ± 0.35 min per patient. The CDSS and manual approach selected the same EN/ONS products in 66.7% of the cases.

**Conclusion:**

Implementing CDSSs in hospitals may streamline workflow, reduce calculation errors and shorten the time needed per patient. The specific CDSS may reduce the time required for EN/ONS planning and may help standardize formula selection in adult hospitalized patients. These findings require confirmation in larger, multicenter studies with predefined clinical and workflow outcomes.

## 1 Introduction

Malnutrition is a common finding in hospitalized and outpatient individuals who receive home‐care treatment [1]. Nutritional support appears to decrease infectious complications, delayed recovery, and incidence of mortality in both patient groups [2]. Μedical nutrition therapy (enteral and parenteral) aims to maintain or restore a healthy nutrition status [2]. The statement “If the gut works use it” [3] has increased the popularity of enteral nutrition (EN) over the last decades. When the gastrointestinal tract is functional, but oral nutrition intake is inadequate or feeding via the oral route is not recommended, nutritional support with enteral formulas is the optimum feeding method [4, 5, 6, 7]. EN support is primarily based on the use of commercial, ready‐to‐use EN formulas, marketed in several countries [8]. These formulas can provide energy and nutrients to fulfil patients' needs [6], while homemade and blended enteral preparations contain fewer micronutrients [9]. Although the literature indicates that the administration of enteral formulas is safe and effective, there are risks associated with medical errors that may yield protein‐energy malnutrition or refeeding syndrome [10, 11, 12]. In addition, several tube feed medication errors may occur [13, 14]. Furthermore, designing an EN plan is a time‐consuming task [15, 16], and dietitian staffing may be inadequate to cover a hospital's needs [17]. However, a standardized feeding protocol and/or specific algorithms are related to optimized administration of nutrients [18, 19].

An increasing number of studies focus on developing and evaluating software tools for nutrition support, aiming to facilitate the medical team's work and achieve a normal nutrition status for patients [17, 20, 21, 22, 23, 24, 25, 26]. Recently, the use of mobile smartphone applications for enteral feeding in the ICU has been reported, which has led to timely and improved delivery of EN [27]. The decreased number of errors, the selection of the appropriate ready‐to‐use nutritional solutions, and the reduced time required to store and process data are the main goals for the majority of the programmes developed [13]. At the same time, previous studies have reported gaps in clinicians' training and confidence regarding EN prescription, highlighting the potential value of structured decision‐support tools in clinical nutrition practice [28]. For these reasons, the development of assisting CDSS tools may facilitate clinical practice.

Thus, the aim of the present work was to narratively review already published CDSS tools for enteral nutrition (EN) and to preliminarily evaluate a locally developed internal‐use CDSS tool designed to calculate patients' nutritional requirements and assist in the selection of EN and oral nutritional supplements (ONS) in adult hospitalized patients.

## 2 Materials and Methods

### 2.1 Literature Review Regarding the Use of CDSS for EN

A narrative literature overview search for CDSS programmes applied in EN was performed from inception (i.e., 1980s) to May 2026 in the PubMed database. The search strategy involved the use of the following terms: “enteral nutrition” OR “artificial nutrition” OR “nutritional supplement” OR “tube feeding” combined with the following terms: “clinical decision support system” OR “Decision Support System” OR “software” OR “program” OR “computer.” The detailed search strategy is outlined in Table 1.

**Table 1:** Search strategy in PubMed.

#1 “enteral nutrition” [MeSH] OR “artificial nutrition” [tiab] or “nutritional supplement” [tiab] or “tube feeding” [tiab] #2 “clinical decision support system” [tiab] or “decision support system” [tiab] or “software” [tiab] or “program” or “computer” [tiab] #1 AND #2

#### 2.1.1 Inclusion and Exclusion Criteria

Inclusion criteria for the studies included were the following: (1) use of a CDSS, (2) use in a hospital setting (3) assistance in EN design or monitoring, and (4) assistance in minimizing drug‐nutrient interactions. Exclusion criteria were: (1) reviews, (2) articles focusing on the data needed to create a CDSS, (3) CDSS for assistance only in parenteral nutrition administration, and (4) CDSS for medical diagnosis. Language restrictions were not applied.

#### 2.1.2 Data Extraction

Two researchers independently (P. D. and P. P.) extracted details from each article, in a pre‐defined Excel sheet. Specifically, the extracted data included information about (a) the first author's name, (b) the year of publication, (c) the name of CDSS (if available), (d) a description of the main functions of CDSS, (e) the study origin, (f) the patients on which the CDSS targets g) mode of administration (i.e., PC, bed‐side platform, smartphone application etc.), (h) intervention details (if available) and (i) results of the intervention.

### 2.2 Development of the Software CDSS Programme

By common clinical practice, data acquired from selected literature were carefully evaluated by licenced dietitians and used to develop databases/algorithms, based on proper predictive equations.

#### 2.2.1 Calculation of Recommended Energy and Macronutrients

Ranges for daily energy recommendations (kcal/kg/day) were determined based on patients' diagnoses. For critically ill patients in ICU, the following weight‐based energy ranges according to the phase of illness are used:

▪ In the acute and initial phase of a critical illness (72–96 h): < 20–25 kcal/kg/day [19]
▪ In the recovery phase: 25–30 kcal/kg/day [19]
▪ Patients severely undernourished: 25–30 kcal/kg/day [19]

Ranges for protein recommendations (g/kg/day) depend on the disease condition. For example, protein requirements in different stages of renal failure (acute renal failure with conservative therapy: 0.6–0.8 g/kg/day, acute renal failure with extracorporeal therapy: 1–1.5 g/kg/day, acute renal failure with continuous renal replacement therapy: up to 1.7 g/kg/day, chronic renal failure before replacement therapy: 0.55–0.60 g/kg/day, chronic renal failure during hemodialysis: 1.2–1.4 g/kg/day, chronic renal failure during peritoneal dialysis: 1.2–1.5 g/kg/day) are automatically calculated after the selection of a patient's disease stage [29]. When published recommendations provided a protein range, the CDSS used the midpoint of that range as the default value to generate an initial estimate of protein requirements. This value could be manually adjusted by the responsible dietitian or nutrition‐support clinician based on clinical judgement and patient‐specific status. Total protein requirements were then automatically expressed in both grams/day and kcal/day. Regarding carbohydrate and fat recommendations, they are calculated as percentages of non‐protein requirements and are automatically transformed into grams and calories.

For patients at risk for refeeding syndrome (patients with chronic alcoholism, prolonged absence of nutrition support, marasmus, kwashiorkor, anorexia nervosa, sepsis, multi‐organ failure, major surgery, cachexia, severe undernutrition, and the elderly) a different approach/algorithm was given by the CDSS. Enteral feeding begins with the provision of 10–20 kcal/kg/day for the first 2–3 days. Potassium, magnesium, calcium, and phosphorus levels are closely monitored and micronutrients are supplemented accordingly [30, 31].

#### 2.2.2 Body Weight Considered in the Calculations

To determine the exact energy and protein requirements body weight is considered. Once all required data in the form “Anthropometric Data” are filled in, the programme automatically gives a patient's body weight (BW) analysis. The analysis includes Current BW (CBW), Adjusted BW (ABW), Dry BW (DBW), Ideal BW (IBW, calculated based on the patient's gender and height), and Predicted Body Weight (PBW). If, for example, a patient has been diagnosed with oedema or ascites, the programme will automatically calculate DBW and that will appear in the field “Calculated Weights.” In the case of sepsis, if PBW is lower than IBW, then PBW is used for the calculation of REE. If PBW is higher than IBW, then IBW is used. If the weight lost over the last 6 months is lower than 10%, PBW is used. If the weight lost is higher than 10%, UBW is used [32, 33]. When oedema is present (ascites or peripheral oedema), DBW is taken into account, and when the patient is of normal BW, CBW.

#### 2.2.3 Formulary

A complete database for all commercially available standard formulas (all available formulas), disease‐specific formulas (all available formulas), and macronutrient nutritional supplements (e.g., carbohydrates, protein, or glutamine compounds) (available formulas) are included in the programme. Nutrition analysis is available for all these formulas and the programme informs the user to what extent a formula covers a patient's nutrient needs. Thus, the user can judge whether a formula is appropriate or inappropriate for a particular patient.

#### 2.2.4 Description of the Software

The developed CDSS includes the following modules: (i) a database module for entering, updating, and storing patient data, (ii) a database module containing several diseases and commercial enteral formulas (iii) a module for editing and updating nutritional components, (iv) a statistics module for patients' data, and (v) a module for arranging new appointments, monitoring and assessing a patient's health progress, upon the completion of each appointment.

Initially, the software requires data entry for each patient (i.e., name, gender, date of birth, occupation, address, and contact details), anthropometric indices (body weight and height), medical data (diagnosis, medical history, medications, laboratory tests), and nursing data (clinical department, admission date, nutrition starting date, catheter type, and date of its placement). Fields for comments on patients' clinical conditions and/or other relevant remarks are available. Some of the previously mentioned input data are mandatory and others are optional.

After diagnosis and determination of the feeding route, the software analyzes input data and calculates nutritional requirements, by implementing evidence‐based equations for energy needs, proteins, carbohydrates, fats, and fluids, on a 24‐h basis. The most suitable standard formula, satisfying each patient's nutritional needs, is selected by the user, from a list of formulas. The programme provides a full nutritional analysis of each formula, and thus the health professional can judge, which is the most appropriate to administer. The programme acts as a guide for the user, providing informative messages and nutrient composition tables.

Each patient's clinical profile and daily progress can be monitored and recorded (including medical history, history of medications, clinical complications, laboratory tests, and other diagnostic tests). The user may intervene and modify data at any time. The programme aims to be a clinician's partner as previously suggested [34].

All programme data can be extracted and analyzed with other statistical computer programmes (Excel, SPSS, Statistica), as a database file format (DBF), and using a “communication” procedure in a readable text format (ASCII).

### 2.3 Preliminary Testing of the CDSS

#### 2.3.1 Study Population

The preliminary evaluation of the CDSS was conducted at IASO Hospital, a private General hospital in Athens, Greece, among adult inpatients. The study included a sample of 27 adult patients who required dietetic assessment and support for EN and/or ONS. Individuals were included in the study if they were ≥ 18 years old, were hospitalized during the study period and had sufficient clinical and anthropometric data available for the estimation of nutritional requirements and the design of an EN/ONS plan.

#### 2.3.2 Study Design

The study design was cross‐sectional. For each patient, the same clinical, anthropometric, biochemical and medical‐record data were used to retrieve the nutrition plan manually and through the CDSS. One trained dietitian with experience in clinical nutrition and EN planning performed both procedures. More specifically, the experienced dietitian (a) manually estimated nutritional requirements and selected the ideal EN plan and (b) entered the data on the CDSS to retrieve automatically nutritional requirements and EN solution. A comparison of the manual and the automatic (CDSS) method for EN planning was performed by a second dietitian who supervised the process, recorded the time required for each procedure and reviewed differences between the two procedures.

#### 2.3.3 Outcomes

The main outcomes were the time needed to complete the EN plan using the manual and the CDSS approach, as well as the agreement of the two approaches regarding EN and/or ONS product selection.

#### 2.3.4 Measurements

Data concerning anthropometric indices (body weight and height, BMI, fat) were collected. Body composition was assessed using Bioelectrical Impedance Analysis (BIA) (Akern BIA 101). Glucose was measured in an automated analyzer (SIEMENS Atellica 4007). All data on diagnosis, clinical condition, medication, and medical history were collected from the medical records. Follow‐up data were collected at 1 month.

#### 2.3.5 Ethical Approval

The Ethics Committee of IASO hospital, in Athens, Greece, examined and approved the study (Approval Code DE31052019). The study was carried out in accordance with the principles of the Helsinki Declaration (1989) and its later amendments.

### 2.4 Statistical Analysis

Normality was tested with the Shapiro‐Wilk test and P‐P plots. Continuous variables were expressed as mean and standard deviation or median and interquartile range, according to their distribution, while categorical variables as counts (n) and percentages (%). Differences in planning time were evaluated using paired samples analysis. Since paired time differences were not normally distributed, the Wilcoxon test was used. The paired mean difference in planning time was reported with its 95% confidence interval (CI). The effect size for the Wilcoxon test was calculated as *r* = |Z | /√N [35]. Agreement between manual and CDSS‐derived EN/ONS product selection was assessed using percentage agreement with 95% CI. The 95% CI for the agreement percentage was calculated using the Wilson score CI method, which is appropriate for binomial proportions in small samples [36]. Baseline and 1‐month follow‐up patient characteristics were compared using paired‐samples *t*‐tests or Wilcoxon tests, as appropriate. Statistical significance was set at *p* < 0.05. All analyses were performed using SPSS 31.0.

## 3 Results

### 3.1 Results on the Literature Review Regarding the Use of CDSS for EN

Fourteen CDSS were identified, the basic characteristics of which are shown in Table 2. The studies were conducted in several countries across the globe, namely Melbourne [37], European countries [13, 17, 38, 39, 40, 41, 42], the United States [24, 27], China [43], the Philippines [44], and Israel [45]. Half of the identified studies were performed in the last decade [13, 27, 41, 42, 43, 45]. Most of the CDSS included the calculation of energy needs and formula recommendations. Although studies were heterogenous, the application of a CDSS resulted in time‐saving [26], increases in dietitian referrals [44], increases in anthropometric values acquisition [44], decreases in nutritional risk [43], faster initiation of enteral feeding [27], increases in patients receiving adequate nutrition [39, 40], and reduction of medical errors [13].

**Table 2:** CDSS systems for enteral nutrition.

References	Year	Country	Name of instrument	Main characteristics	Patients	Mode of administration	Comparison studies
Wilson et al. [37]	1983	Australia	NICUDAMAS 1 (Neonatal Intensive Care Unit DAta MAnagement System)	Recording body weight; fluid intake, energy, macronutrients, sodium and calcium intake (total, oral enteral, and parenteral)	Critically ill neonates	PC	NA
Dartois et al. [38]	1989	France	NA	Prescribed diet (energy, micro, and macronutrients) from proposed foods and enteral supplements	Children with renal problems	PC	NA
Quintaliani et al. [26]	1994	Italy	NA	Calculation of nutritional needs, update on available supply, enteral product or combination, mixtures recommendation, choices modification, outputs	Patients with renal problems	PC	Saving time in decision making (3–5 min for each patient) (no data available)
Arbeloa et al. [17]	1995	Spain	NA	Computer programme of artificial nutrition	Adults, critically ill patients	PC	NA
Schloerb et al. [24]	2000	US	Electronic Parenteral and Enteral Nutrition (EPEN), Kansas University medical centre	Calculation of energy, macro‐ and micro‐ nutrient needs, optimum parenteral and EN support	Adults, children	PC	NA
Llido et al.[44]	2006	Philippines	NA	Nutrition screening, lists of malnourished patients, and calculation of needs and formulas for either nutritional requirement or parenteral and/or enteral formulation. patient status report generation	Adults, children, critically ill patients	PC	↑ Data entry of weight and height (90% vs. 30%) ↑ dietitians referrals (83% vs. 38% for patients and 99% vs. 10.3% for critically ill patients
van Schijndel et al.[39]	2009	the Netherlands	NA	Advice on type and amount of nutritional formula	critically ill patients	PC	↑ Patients receiving adequate nutrition from 30.2% to 56.5% ↓ICU length of stay
Conseil et al.[40]	2013	France	NA	Energy calculation, nitrogen and non‐nitrogen calories (spreadsheet)	critically ill patients	PC	↑Calorie and nitrogen intake ↓ ICU‐acquired infection (59% vs. 41%), nonsignificant difference in mortality/ICU length of stay.
Villalobos Gámez et al. [41]	2014	Spain	INFORNUT®	Nutritional screening, Malnutrition risk, nutritional risk, diagnostic codes of malnutrition, cost of EN	adults	PC	NA
Siquier Homar et al [42]	2015	Spain	NA	Nutritional screening, indirect height determination, determination of energy needs, diet prescription, traceability system of raw materials, detection of errors in parenteral nutrition, report generation	adults	PC	NA
Mahmood et al. [27]	2019	United States	Tube Feed	Recommendation of EN formulas (two choices)	Critically ill adults	Smartphone/device application	*n* = 160 pre‐application (control) *n* = 216 post‐application ↓ % patients with delayed initiation of EN (37.5% vs. 61.2% *p* < 0.01). ↓ mean time to initiate EN (31.9 vs. 44.5 h). No effect on length of stay and survival rates.
Wasylewicz et al. [13]	2021	Netherlands	Gaston Pharma (version 2.8.2.100, Gaston Medical, Eindhoven, the Netherlands)	Detection of feeding tube related medication errors	Adults	PC	*n* = 38 pre‐intervention (control) *n* = 43 intervention The incidence of feeding tube‐related medication errors before the intervention was 0.15 (95% CI, 0.07–0.23) vs. 0.02 (95% CI, 0.00–0.04) in the intervention phase.
Hu et al. [43]	2022	China	NA	“Internet + medical” training platform for nurses basic database, management, training, resource database, remote consultation, case discussion module, evaluation	Adults	Internet, PC	*n* = 40 control *n* = 40 intervention ↓ nutritional risk (NRS2002) after programme implementation (2.89 ± 0.75 vs. 3.25 ± 0.82). Positive effects on hematological indices.
Kagan et al. [45]	2023	Israel	smART+ Platform (ART MEDICAL, Netanya, Israel)	Platform including tube location, gastric intolerance, resting energy expenditure measurement, and selection of enteral formula. Detection of feeding interruptions and compensation.	Critically ill adults	Sensor‐based bed‐side platform	*n* = 48 intervention with the CDSS *n* = 50 control comparison in day 2 and day 14. ↓ Mean feeding deviation (10.5% ± 13 for smart + group vs. 34.3% ± 18 for the control [*p* < 0.0001]). The percentage of days within 90‐110% of the target was 75.7% for smart+ vs. 23.3% for control (*p* < 0.0001) ↓ length of stay was reduced (mean 10.4 vs. 13.7 days) ↓ length of ventilation was reduced (mean 9.5 vs*.* 12.8 days)

Abbreviations: NA, not available; PC, personal computer.

### 3.2 Results Regarding the Preliminary Evaluation of the CDSS

#### 3.2.1 Basic Characteristics of Patients and 1‐Month Follow‐up

The study included 27 adult inpatients requiring EN and/or ONS planning. The most common feeding‐related condition was gastrostomy, present in 12 patients. Dysphagia was recorded in four patients, while four patients had gastrointestinal surgery or stoma‐related conditions, including colectomy, ileostomy, colostomy or bowel resection. Cancer‐related conditions were present in seven patients (i.e., pancreatic, rectal, laryngeal cancer, and lymphoma). Six patients with gastrostomy also had obesity as a recorded comorbidity. Other conditions included liver transplantation, gastrectomy with total splenectomy, malnutrition, inadequate oral intake, diarrhea and weakness (Table S1). The characteristics of patients included in this study are presented in Table 3.

**Table 3:** Baseline characteristics of the *N* = 27 adult inpatients.

Characteristics	Mean ± standard deviation (or otherwise stated)
Females, *n* (%)	15 (55.5)
Gastrostomy, *n* (% yes)	12 (44.4)
Cancer, *n* (% yes)	7 (25.9)
Weight (kg)	79.02 ± 20.85
BMI (kg/m^2^)	27.97 ± 6.17
Body Cell Mass (%)	30.6 ± 8.4
Fat (kg)	27.99 ± 12.66
Fat (%)	33.95 ± 11.03

*Note:* Results are presented as mean values ± standard deviation for continuous variables and as counts (*n*) and frequencies (%) for categorical characteristics.

Abbreviation: BMI, body mass index.

Several anthropometric characteristics and biochemical parameters at baseline and 1‐month follow‐up after CDSS‐derived EN/ONS regimen are presented in Table 4. It was revealed that fat mass (*p* = 0.013) and fat mass (%) (*p* = 0.031) were lower at follow‐up compared with baseline. No statistically significant changes for weight, BMI and glucose levels were observed (all *p*'s > 0.05).

**Table 4:** Basic anthropometric characteristics and biochemical parameters at baseline and 1‐month follow‐up after CDSS‐derived EN and/or ONS regimen for the total sample (*N* = 27).

Characteristics	Baseline (mean ± SD)	Follow‐up (mean ± SD)	*p* value
Weight (kg)	79.02 ± 20.85	77.40 ± 19.72	0.078
BMI (kg/m^2^)	27.97 ± 6.17	27.09 ± 5.39	0.118
Fat (kg)	27.99 ± 12.66	25.61 ± 11.65	**0.013**
Fat (%)	33.95 ± 11.03	31.77 ± 10.14	**0.031**
Glucose (mg/dL)	105.93 ± 34.70	101.25 ± 21.83	0.185

*Note:* Results are presented as mean ± standard deviation (SD). *p* values were derived from paired‐samples *t*‐tests comparing baseline and 1‐month follow‐up values. Statistical significance was set at *p* < 0.05, marked with bold.

Abbreviations: BMI, body mass index; EN, enteral nutrition; ONS, oral nutritional supplements.

#### 3.2.2 Outcomes of CDSS Utilization

A significant difference was noted between the manual and the computerized method concerning the time needed to design the diet plan (*p* < 0.001). The completion of all necessary procedures for the manually derived EN/ONS plan required 540.63 ± 12.6 s (or 9.01 ± 0.21 min). In contrast, the CDSS‐derived plan required 240.81 ± 21.3 s (or 4.01 ± 0.35 min) (Figure 1). The mean paired reduction in planning time was 5.00 ± 0.46 min per patient (95% CI: 4.81−5.18 min) (or mean ± SD: 299.81 ± 27.64 s, 95% CI: 288.88−310.75 s) per patient, corresponding to an approximately 55.5% reduction. Since the paired time differences were not normally distributed, the Wilcoxon test was used. Manual planning time was higher than CDSS‐derived planning time in all 27 cases and the reduction was statistically significant (*Z* = −4.542, *r* = 0.87, *p* < 0.001).

**Figure 1:**
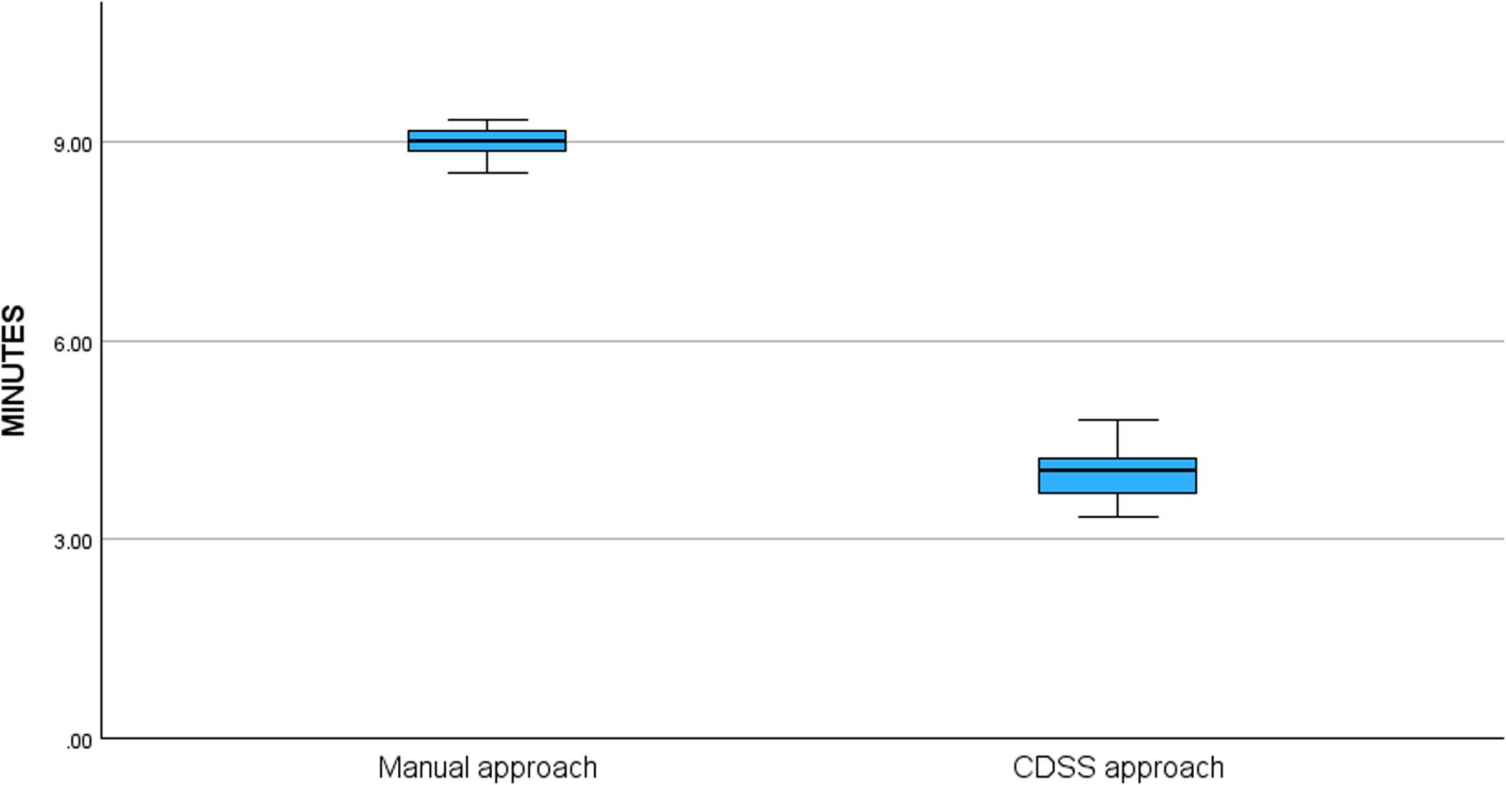
Time needed for decision making for dietitians (manual approach) and CDSS system (CDSS approach). CDSS, Clinical Decision Support System.

Regarding the level of agreement between the manual and CDSS‐approach, it was observed that both approaches selected the same EN/ONS product in 18 out of 27 patients (66.7%) (95% CI: 47.8–81.4%). In 9 out of 27 cases (33.3%), the choice of manual enteral solution would have been different from that recommended by the programme. Details on specific choices of dietitians and the CDSS are shown in Table S1.

## 4 Discussion

In the present pilot study, the locally developed for internal‐use CDSS, designed to calculate patients' nutritional requirements and assist in the selection of EN and ONS was tested in a sample of 27 adult patients admitted to a private General Hospital in Athens. The manual and the automatic (software) approach for EN planning were compared regarding the time needed to complete the EN plan and the level of agreement of the two approaches regarding EN and/or ONS product selection. It was found that compared with the manual approach, CDSS‐derived planning reduced the time required for diet‐plan design by 55.5%, that is, approximately 5 min less time per patient. Agreement between the CDSS and the manual approach in EN/ONS product selection was observed in 66.7% of cases. These findings suggest that the CDSS may improve workflow efficiency and support standardization in EN/ONS planning. Finally, literature screening for other software tools available for EN in adults revealed that few articles have been published evaluating the potential benefits of computer‐assisted EN.

The software described herein reduces time needed and the occurrence of prescription errors, regarding the formulas selected and the amounts administered. In previous studies, an implementation of a computerized programme for patients with kidney problems was related to a 3–5 min reduction in the time needed for calculations for each patient [26]. Several CDSS programmes have been also created for patients [46] but only ~24% of them include dietary recommendations [47]. It has been suggested that future CDSS should focus on screening, diagnosis, and management of diseases [47]. Hopefully, the developed CDSS ensures the implementation of the nutrition care process [48, 49]. The clinical usefulness of the CDSS used in the present study (without EN supplements) was recently demonstrated in patients with cancer [48], auto‐immune diseases [50, 51], polycystic ovaries syndrome [52], and women in pregnancy [53].

The particular software may positively impact on the daily workload of healthcare professionals, since it provides a large database of commercially available EN formulas. Thus, programme users need neither to search for available enteral formulas nor make calculations to find which one best satisfies a patient's nutritional needs. Consequently, the particular software facilitates the decision‐making process for the nutrition support team. The database containing enteral formulas may be constantly updated, so that new products are incorporated and old ones or outdated ones are deleted. It should be noted though that formula availability, product names and nutrient composition may vary by country, healthcare setting and over time. Therefore, local product databases should always be updated before implementation in other settings. The whole programme constitutes an electronic health record (EHR) of patients, who have received EN treatment and have been monitored for a specific period. All this information can be used for statistical analysis, conducted by healthcare professionals or pharmaceutical companies.

The programme was not developed to substitute clinical diagnosis by healthcare providers, but to assist them in calculations, storage of patients' data, selection of the proper EN formulas, patients' monitoring and appointment planning especially for outpatient individuals. Moreover, the software may also be a useful teaching tool for resident medical, nursing, and pharmacy personnel. It allows an individual to evaluate how changing a formula characteristic, such as caloric density or protein content, may influence the nutrient composition and volume infused. The programme may be used to try various “what‐if scenarios” and teach the proper prescription of enteral formulas.

Limitations of this study are that it is a single‐centre trial as other studies [27]. To achieve optimum outcomes with this software, some prerequisites should be met. Transcribing information into the programme should be done with great care to avoid errors (e.g. prescription errors, transcription/interpretation errors, distribution errors, and administration errors). As the system is very sensitive to typing errors when searching in the drug database, features that recognize words and text, making the programme much less sensitive to typing errors, still need to be integrated. Moreover, for best results patient data could be taken from the EHR. The nutritional support team should be well‐versed, in how to use the programme efficiently; the members should have nutritional knowledge and all relevant information regarding patient characteristics, for example, diagnosis, disease state, and biochemical data. The software will be constantly updated to incorporate changes in research and practice guidelines. Also, the developed programme requires dietitians to have access to a computer, which is rarely located at the bedside or instantly at their disposal. New developments, such as wireless communication systems and handheld computers, may overcome this problem. In case technical changes are needed in the evaluation process of the software, these can be easily incorporated in a revised version of the software. Also, future developments may include interfacing the software with other databases of the hospital information systems, for example pharmacy, so that the prescribed EN formulas can be ordered automatically. In the future, artificial intelligence algorithms may also be incorporated [54].

In conclusion, implementing CDSSs in hospitals may generally streamline workflow, reduce calculation errors and shorten the time needed per patient. The described software may support EN/ONS planning in hospitalized adults by reducing the time required per patient and by promoting a more standardized approach to product selection. A larger scale application is required to fully assess the effectiveness of the software in assisting the hospital management in satisfying each patient's nutritional needs.

## Author Contributions

**Panos Papandreou:** conceptualization, investigation, writing – original draft preparation. **Paraskevi Detopoulou:** conceptualization, investigation, methodology, validation, formal analysis, writing – review and editing, writing – original draft preparation, supervision. **Alexandra Foscolou:** validation, formal analysis, writing – review and editing. **Aristea Gioxari:** validation, writing – review and editing. All authors have read and agreed to the published version of the manuscript.

## Funding

The authors have nothing to report.

## Conflicts of Interest

The authors declare no conflicts of interest.

## Supporting information


Supporting File

## Data Availability

The data that support the findings of this study are available on request from the corresponding author. The data are not publicly available due to privacy or ethical restrictions. Data available on request due to privacy/ethical restrictions.
