# Supplementary material for: Clinical Decision Support for Adult Enteral Nutrition: Literature Overview and Preliminary Hospital‐Based Evaluation
Source: J Eval Clin Pract. 2026 Oct 12;32(7):e70612. doi: 10.1111/jep.70612 (PMC13657400; doi:10.1111/jep.70612)
Supplement: Supplementary file 1 [file JEP-32-0-s001.docx]

| Supplementary Table 1. Agreements and differences between CDSS- and manual derived product selection at the patient level. | | | |
| --- | --- | --- | --- |
| Patient | **Clinical condition / feeding-related indication** | **CDSS EN/ONS product** | **Manual EN/ONS product** |
| 1 | Pancreatic Cancer with Metastasis - Weakness - Disease | SUPPORTAN DRINK | CLINUTREN HP/ HC |
| 2 | Gastrectomy - Total Splencectomy | FRESUBIN ORIGINAL | FRESUBIN ORIGINAL |
| 3 | Ca Rectum X/O Colon - Removal 20 Cm Large Intestine - Colostomy | ISOSOURCE STANDARD | ISOSOURCE STANDARD |
| 4 | Liver Transplantation | FRESUBIN HEPA | SUPPORTAN DRINK |
| 5 | Larynx Cancer - Gastrostomy | SUPPORTAN | SUPPORTAN |
| 6 | Gastrostomy - Obesity | DIBEN | DIBEN |
| 7 | Total Colectomy - Ileostomy Convergence - For small weight loss | ISOSOURCE STANDARD | DIBEN |
| 8 | Malnutrition - Inability to adequately Eat - Diarrhea | FRESUBIN ORIGINAL DRINK | FRESUBIN ORIGINAL DRINK |
| 9 | Gastrostomy | ISOSOURCE STANDARD | ISOSOURCE STANDARD |
| 10 | Total Colectomy - Ileostomy - Restoration | ISOSOURCE STANDARD | DIBEN |
| 11 | Dysphagia | FRESUBIN CRÈME | FORTIMEL CRÈME |
| 12 | Weakness | CLINUTREN HP/ HC | CLINUTREN HP/ HC |
| 13 | Gastrostomy | ISOSOURCE PROTEIN | ISOSOURCE PROTEIN |
| 14 | Gastrostomy | ISOSOURCE PROTEIN | ISOSOURCE PROTEIN |
| 15 | Dysphagia - Ca Pancreas | FRESUBIN CRÈME | FORTIMEL CRÈME |
| 16 | Gastrostomy | SUPPORTAN | SUPPORTAN |
| 17 | Dysphagia | FRESUBIN CRÈME | FORTIMEL CRÈME |
| 18 | Gastrostomy - Obesity | DIBEN | DIBEN |
| 19 | Gastrostomy | ENSURE PLUS HN | ENSURE PLUS HN |
| 20 | Gastrostomy - Obesity | DIBEN | DIBEN |
| 21 | Gastrostomy | SUPPORTAN | SUPPORTAN |
| 22 | Gastrostomy - Obesity | DIBEN | DIBEN |
| 23 | Dysphagia | FRESUBIN CRÈME | FORTIMEL CRÈME |
| 24 | Total Colectomy | ISOSOURCE STANDARD | ISOSOURCE STANDARD |
| 25 | Lymphoma | SUPPORTAN DRINK | CLINUTREN HP/ HC |
| 26 | Gastrostomy - Obesity | DIBEN | DIBEN |
| 27 | Gastrostomy - Obesity | DIBEN | DIBEN |

Green shading indicates agreement between approaches; red shading indicates product-selection discrepancy. Commercial product names are presented to describe the products available and used in the hospital setting during the study period. CDSS: Clinical Decision Support System; EN: enteral nutrition; ONS: oral nutritional supplements.
